# Application of Hybrid Platelet Technology for Platelet Count Improves Accuracy of PLT Measurement in Samples from Patients with Different Types of Anemia

**DOI:** 10.3390/jcm14155401

**Published:** 2025-07-31

**Authors:** Małgorzata Wituska, Olga Ciepiela

**Affiliations:** 1 Laboratory of Central Teaching Hospital, University Clinical Center of Medical University of Warsaw, 02-091 Warsaw, Poland; malgorzata.wituska@uckwum.pl; 2 Department of Laboratory Medicine, Medical University of Warsaw, 02-091 Warsaw, Poland

**Keywords:** hybrid PLT, fluorescent PLT, anemia, evaluation

## Abstract

**Background:** Reliable platelet (PLT) measurement is crucial for the accurate diagnosis of thrombocytopenia. Several methods exist for automated PLT counting, including the impedance method (PLT-I), as well as optical and fluorescence methods (PLT-F). The impedance method is cost-effective but susceptible to interference from small red blood cells and schistocytes. In contrast, fluorescent assessment offers higher specificity but is more expensive, as it requires additional dyes and detectors. Hybrid platelet counting (PLT-H) combines impedance with measurements from the leukocyte differentiation channel and is available without additional cost. **Aim:** The aim of this study was to evaluate the accuracy of hybrid PLT counting in anemic samples. **Methods:** In this retrospective study, PLT counts from 583 unselected anemic samples were analyzed using two different analyzers: the Sysmex XN3500, equipped with fluorescent PLT-F technology, and the Mindray BC6200, which uses both impedance (PLT-I) and hybrid (PLT-H) technologies. Agreement between PLT-I and PLT-F, as well as between PLT-H and PLT-F, was assessed using Bland–Altman plots. Correlation between the methods was evaluated using the Pearson correlation coefficient. **Results:** The hybrid method demonstrated better accuracy in PLT counting compared to the impedance method. Correlation between PLT-H and PLT-F was excellent, ranging from 0.991 to 0.999. In thrombocytopenic samples (PLT < 50 G/L), the hybrid method also provided more reliable PLT counts than the impedance method, reducing the number of falsely elevated PLT results by nearly fivefold. **Conclusions:** Hybrid platelet counting yields more accurate results than the impedance method in anemic samples and shows excellent correlation with the fluorescence method.

## 1. Introduction

Anemia is a major health issue affecting an extremely high number of people worldwide. It is estimated that more than 25% of the human population suffers from decreased hemoglobin concentration of different etiologies [1]. Its frequency is even higher in inpatient settings—data show that preoperative anemia alters up to 40% of patients qualified for major surgery, and even up to 90% patients after major surgery experience blood loss leading to anemia [2]. Several causes leading to anemia may affect the production, the life span, and the destruction of other blood cells, including platelets (PLT). Reactive thrombocytosis was found to be associated with iron deficiency anemia [3,4]; thrombocytopenia often occurs in patients with hematological malignancies [5], and severe infections, including sepsis, may lead to a decrease in platelet number due to activation of disseminated intravascular coagulation [6]. On the other hand, different pathologies in the structure of red blood cells or even leukocytes may interfere with the platelet count—it has been reported that leukocyte residuals, cryoglobulinemia, or microcytosis may affect PLT count, falsely elevating their number [7,8,9,10]. The interference, which is mainly reported when platelets are counted using the impedance method, is caused by the cells or particles in peripheral blood that reflect the platelet size [9]. The impedance method used for assessing the platelet count (PLT-I) is the most widely used and the cheapest tool, in which automated hematological analyzers are equipped with. It was the first automated PLT counting method introduced and significantly improved the precision of thrombocyte evaluation; however, its proneness to interferences forced development of more precise methods for PLT evaluation. Now, additional methods—optical (PLT-O) and fluorescent (PLT-F)—allow for the measurement of platelets automatically and even more accurately, but the flow cytometric method using monoclonal antibodies against CD41 or CD61 is considered to be the reference [11].

Recently, a hybrid method of platelet evaluation was introduced (PLT-H). The principle of this method is based on counting small platelets (of less than 10 fL) using an impedance channel and larger platelets in differential channels using flow cytometry. Combining these two channels is supposed to provide a more accurate platelet count without using any additional reagents, which should be cost-effective and more precise and reliable than using only the impedance method [12].

Accurate platelet (PLT) counting is essential for assessing bleeding risk, guiding transfusion decisions, and informing therapeutic strategies [12]. Erroneous PLT results may lead to inappropriate clinical management and adversely affect patient outcomes. In most inpatient and outpatient settings, laboratory reports provide only a numerical PLT value, without indicating whether the result may be affected by analytical interferences. As such, it is the responsibility of the laboratory to implement the most accurate available method for PLT measurement to ensure the delivery of reliable and clinically meaningful results.

Given the complexity of blood components in patients with anemia and its potential impact on platelet counts, this study aims to investigate the clinical utility of the PLT-H technology for platelet counting in anemic samples and to compare it with the traditional impedance method.

## 2. Materials and Methods

### 2.1. Included Samples

Complete blood counting was performed in 583 non-selected anemic samples (hemoglobin concentration of less than 11.5 g/dL) collected to tubes containing K2 EDTA using two different analyzers: Sysmex XN3500 (Kobe, Japan) equipped with fluorescent PLT-F technology, and Mindray BC6200 (Shenzhen, China) equipped with PLT-H technology. Both analyzers were also able to measure PLT count with the impedance and optical methods. All subsequent tests were completed within 4 h after blood collection. Samples were divided into 9 categories, based on the etiology of anemia that was developed in patients. The number of samples included in each specific group is shown in Table 1. Six samples were disqualified due to detected EDTA-dependent PLT aggregation. All samples were tested within a routine diagnostic protocol—first on Mindray BC6200 and then with Sysmex XN3500 as a reflex test due to flagged abnormalities in hemoglobin or platelet measurements, so the patient’s informed consent was waived.

### 2.2. Impedance Platelets Measurement

To compare different methods, platelets were counted using the impedance method on a Mindray BC6200 hematology analyzer (PLT-I) (Shenzhen Mindray Bio-Medical Electronics Co., Ltd., Shenzhen, China). The principle of the methodology is based on counting cells by measuring the resistance across two electrodes as cells pass through an aperture, without detecting the actual type of cells [13]. Since the detector for PLT measured by the impedance method is designed to measure particles of a defined size, the final result my by affected by micro-red blood cells, schistocytes, or other cellular debris present in the sample [14].

### 2.3. Hybrid Platelet Measurement

**Figure 1:**
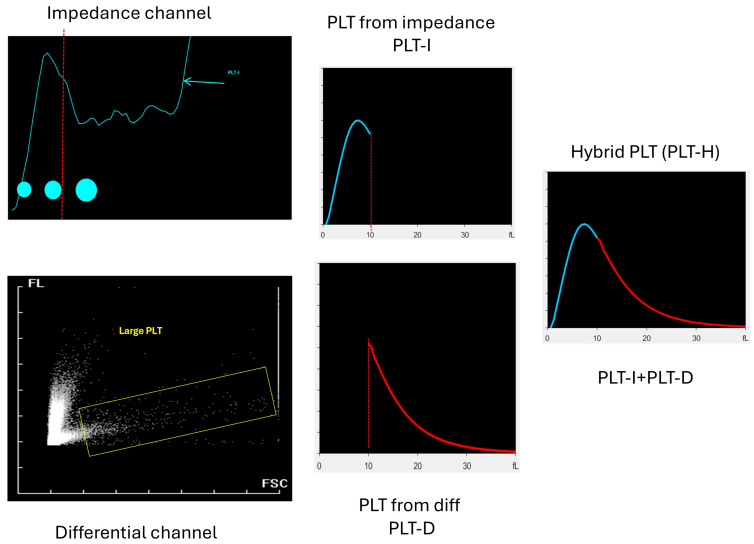
The principle of hybrid platelet measurement. PLTs that are 10 fL and smaller are counted with the impedance method (blue line), and PLTs with a volume exceeding 10 fL are measured in a differential channel using flow cytometry (red line). The final result of PLT-H summarizes the PLT count from both channels.

The second method for platelet counting was a hybrid method using the Mindray BC6200 hematology analyzer. The hybrid method, PLT-H, combines both impedance and a differential channel to count platelets in the analyzed sample. The principle of this method is based on cell size—impedance is used for measurement of particles up to 10 fL, and the differential channels is used for counting platelets where the size exceeds 10 fL. Then, the results obtained from both channels are summarized. A schematic presentation of the methodology is presented in Figure 1. It allows for reducing interferences from microRBCs and schistocytes, which are bigger than 10 fL but not included in the differential analysis due to application of an RBC lysing agent. On the other hand, it allows for including large platelets in the analysis, which are out of reach of impedance measurement due to cell size limitations [14,15].

### 2.4. Fluorescent Platelet Measurement

As a reference method for platelet counting, fluorescent-based PLT detection on the Sysmex XN3000 device was used (PLT-F). Its principle is based on fluorescent flow cytometry, which detects platelets bound to oxazine, a fluorescent RNA staining dye. Its accuracy is highly comparable to flow cytometric assessment based on detection of CD41/CD61 with monoclonal antibodies [16,17].

### 2.5. Statistical Analysis

This study employed ordinary linear regression and Bland–Altman analysis to evaluate the anti-interference capability of the PLT-H technology in various types of anemic samples. Ordinary linear regression was used to assess systematic and proportional bias. Bland–Altman plots were used to visually display the bias and calculate the 95% limits of agreement between compared methods—PLT-I vs. PLT-F and PLT-H vs. PLT-F. The correlation between the two methods was described using the Pearson correlation coefficient. All statistical analyses and graph plotting were performed using GraphPad Prism 10.0 and Origin 2024, with *p* < 0.05 considered statistically significant.

## 3. Results

**Figure 2:**
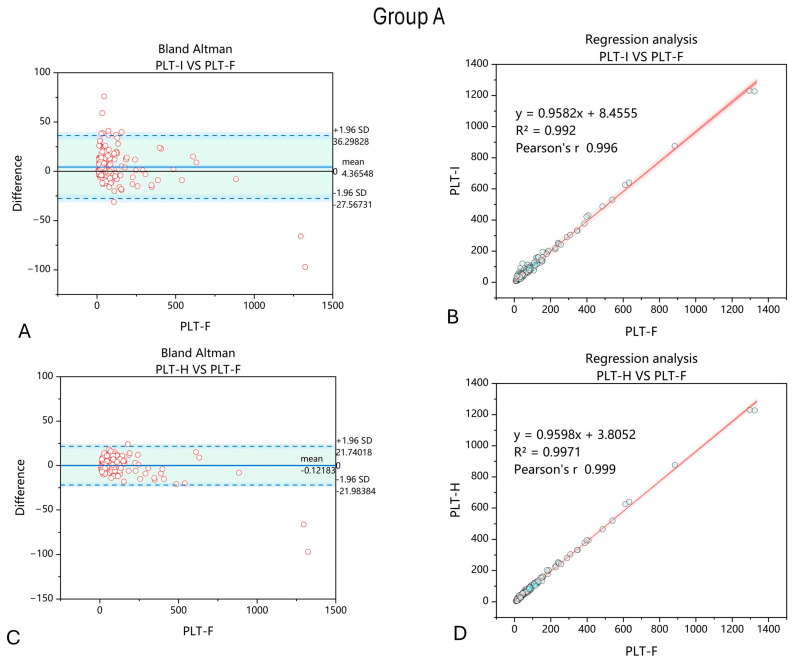
Comparison of PLT-I and PLT-H methods with regard to PLT-F measured in samples collected from patients with anemia due to non-hematological and metabolic disorders. Bland–Altman chart for PLT-I vs. PLT-F method (**A**) shows wider dispersion of results with a mean difference of 4.36 compared to −0.12 obtained when comparing PLT-H and PLT-F methods (**C**). The correlation of the obtained results increased when the PLT-H method on the Mindray BC6200 device was applied (**D**) compared to PLT-I (**B**). *n* = 197; PLT-I—platelet count measured with impedance method; PLT-H—platelet count measured with hybrid method; PLT-F—platelet count measured with fluorescence method.

The first analyzed group consisted of 197 samples from patients with anemia due to non-hematological inflammation of metabolic causes. When comparing the results of PLT count using the impedance and hybrid methods, we observed improvements in the correlation between PLT-F and PLT-H compared to PLT-I. Moreover, the dispersion of differences between PLT-F and the other two methods of PLT counting were smaller when the PLT-H method was applied (Figure 2).

**Figure 3:**
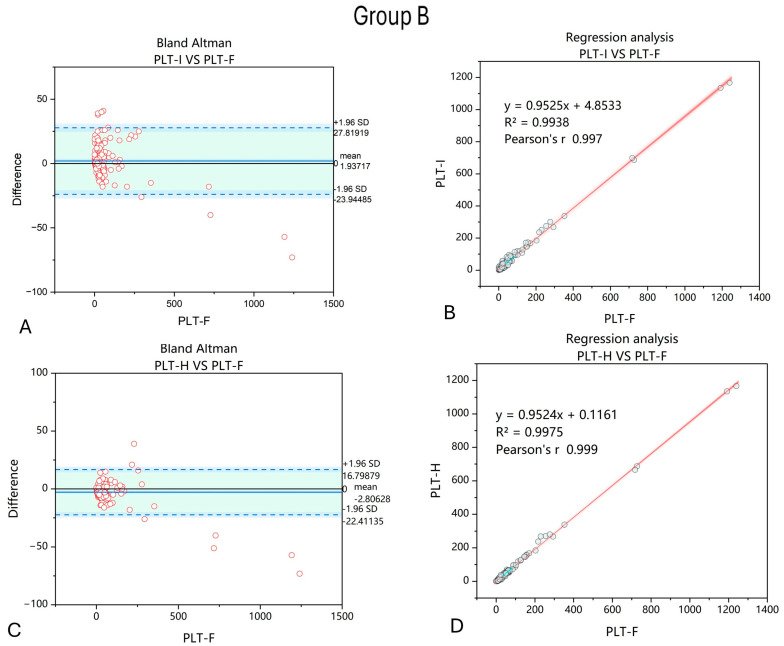
Comparison of PLT-I and PLT-H methods with regard to PLT-F in samples collected from patients with anemia due to hematological cancers and other disorders affecting WBC, RBC, and PLT production. Bland–Altman chart for PLT-I vs. PLT-F method (**A**) shows wider dispersion of results, with a mean difference of 1.94 compared to −2.81 obtained comparing the PLT-H and PLT-F methods (**C**). The correlation of the obtained results increased when the PLT-H method on the Mindray BC6200 device was applied (**D**) compared to PLT-I (**B**). *n* = 193; PLT-I—platelet count measured with impedance method; PLT-H—platelet count measured with hybrid method; PLT-F—platelet count measured with fluorescence method.

The second group consisted of 193 samples from patients with anemia caused by affected RBC production due to hematological neoplasms. Similarly to the first group, applying the PLT-H method for platelet counting improved the correlation between PLT-F and the other PLT counting method. Also, the dispersion of differences between PLT-F and PLT-H was decreased compared to between PLT-F and PLT-I, with a reduced number of outliers (Figure 3).

**Figure 4:**
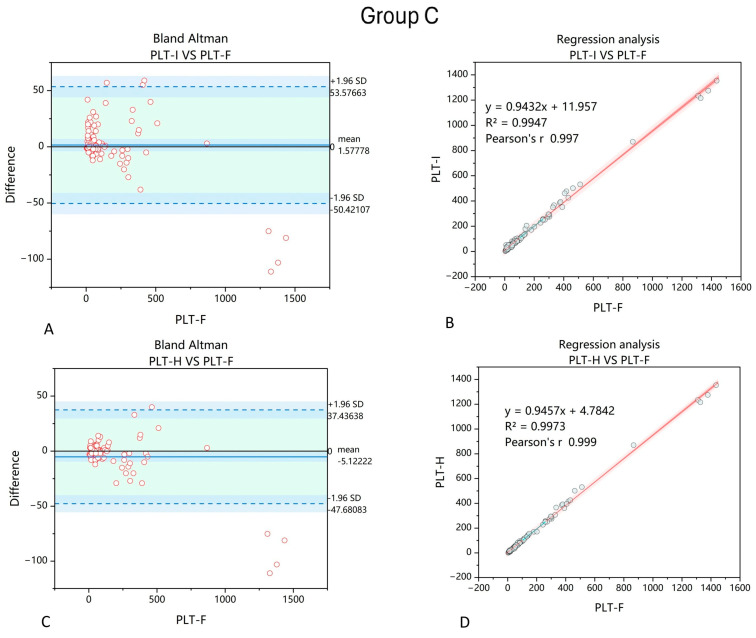
Comparison of PLT-I and PLT-H methods with regard to PLT-F in samples collected from patients with anemia due to non-hematopoietic-related solid tumors. Bland–Altman chart for PLT-I vs. PLT-F method (**A**) shows wider dispersion of results, with a mean difference of 1.58 compared to −5.12 obtained when comparing the PLT-H and PLT-F methods (**C**). The correlation of the obtained results increased when the PLT-H method on the Mindray BC6200 device was applied (**D**) compared to PLT-I (**B**). *n* = 90; PLT-I—platelet count measured with impedance method; PLT-H—platelet count measured with hybrid method; PLT-F—platelet count measured with fluorescence method.

The next group of samples were collected from patients suffering from anemia due to cancer cachexia in the course of solid tumors not affecting bone marrow (*n* = 90). Similarly to the other two groups, applying the PLT-H method also improved PLT counting when comparing to values obtained by the PLT-F method (Figure 4).

Surprisingly, when analyzing the samples with microcytic anemia (*n* = 39), the results obtained with PLT-I and PLT-H were more comparable than in the other anemic groups. Pearson’s coefficients in both cases were 0.996, and the mean values when comparing between PLT-F and PLT-I and between PLT-F and PLT-H were −3.13% and −8.38%, respectively.

**Figure 5:**
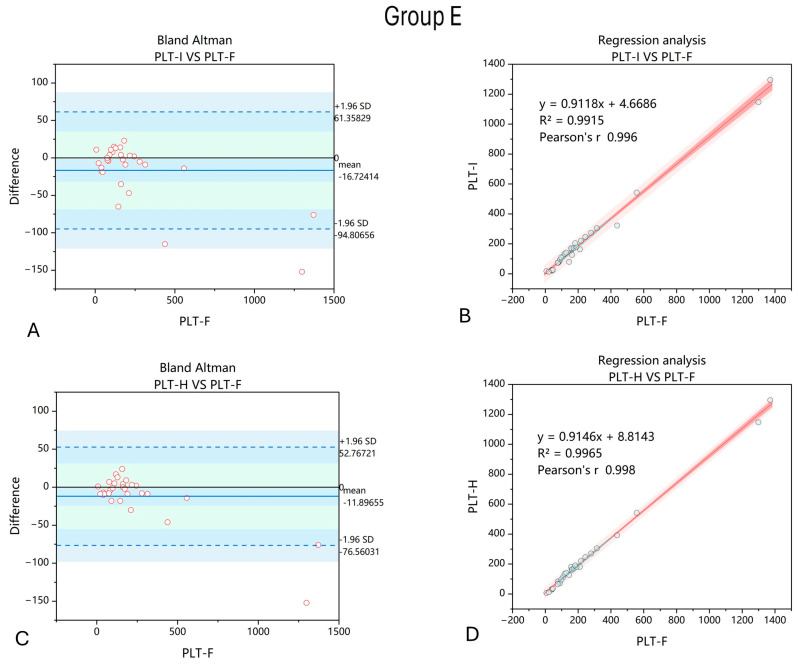
Comparison of PLT-I and PLT-H methods with regard to PLT-F in samples collected from patients with non-inherited hemolytic anemia associated with cardiovascular events. Bland–Altman chart for PLT-I vs. PLT-F method (**A**) shows wider dispersion of results, with a mean difference of −16.72 compared to −11.90 obtained comparing the PLT-H and PLT-F methods (**C**). The correlation of the obtained results increased when the PLT-H method on the Mindray BC6200 device was applied (**D**) compared to PLT-I (**B**). *n* = 29; PLT-I—platelet count measured with impedance method; PLT-H—platelet count measured with hybrid method; PLT-F—platelet count measured with fluorescence method.

PLT-H showed better correlation with PLT-F than PLT-I in samples with anemia caused by cardiovascular causes (artificial valves). Also, the dispersion of differences between PLT-F and PLT-I and between PLT-F and PLT-H were smaller for PLT-H (Figure 5).

**Figure 6:**
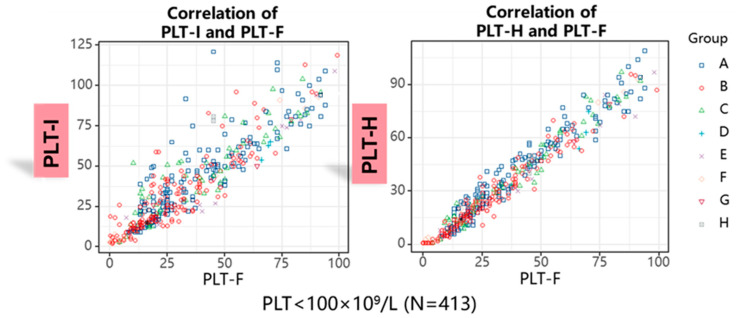
Comparison of platelet count obtained by PLT-I and PLT-H in samples with thrombocytopenia (PLT-F < 100 G/L). The correlation coefficient for PLT-I vs. PLT-F was 0.902, and for PLT-H vs. PLT-F, it was 0.976.

Next, we decided to analyze how applying the PLT-H method influences PLT counting in anemic samples with thrombocytopenia. From the study group, there were 413 samples extracted with a PLT-H count of less than 100 G/L. We showed better correlation in terms of PLT count between the hybrid and fluorescence methods than between the impedance and fluorescence methods (Figure 6).

Further, we decided to analyze how the PLT counting method affects the deviation from the results obtained with the PLT-F method. According to the 2025 Clinical Laboratory Improvement Amendments proposed acceptance limits for proficiency testing, a PLT counting result floating within 25% of the target value is acceptable [18]. In our study, we proposed acceptable criteria for the PLT count deviation for our reference of between 20 and 50%, depending on PLT count (Table 2).

We observed that the results of 22.28% samples (*n* = 92) obtained with PLT-I exceeded the established acceptable deviation, compared to only 2.66% samples (*n* = 11) obtained with PLT-H. Then, we took a step further and decided to focus on samples with a PLT count of less than 50 G/L (*n* = 305).

**Figure 7:**
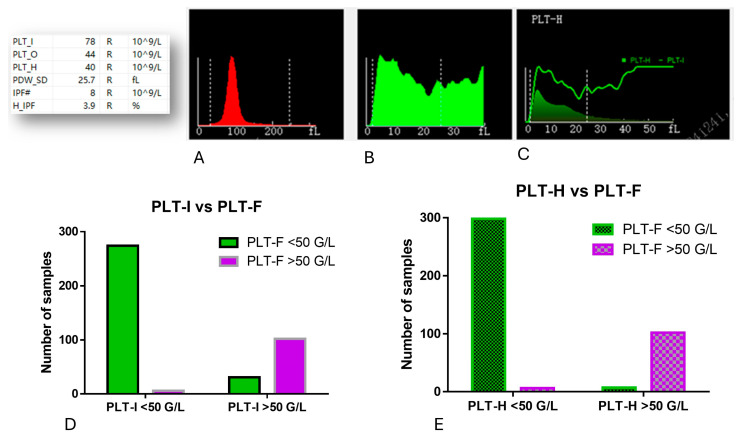
Histograms showing platelet count measured with impedance (**B**) and hybrid methods (**C**) in the presence of microRBCs (**A**) obtained with the Mindray BC6200 analyzer when analyzing thrombocytic samples. The accuracy of PLT counting with the PLT-I and PLT-H methods compared to PLT-F technology in samples with PLT-F counts of less than 50 G/L are shown in (**D**,**E**), respectively. IPF#—number of immature platelets.

We wanted to estimate how many samples with a critically low PLT count measured with the PLT-F method would be similarly qualified as <50 G/L with the PLT-I and PLT-H methods. As the impedance method is based on cell size, microRBCs and schistocytes might be falsely recognized by the analyzer as PLTs (Figure 7A–C). We found that PLT-I falsely increased the PLT count in samples with thrombocytopenia—among all 305 samples measured with the PLT-F method, in 31 (10.16%), PLT-I showed results over 50 G/L. On the other hand, PLT-H gave results >50 G/L only in seven samples (2.29%) (Figure 7D).

**Figure 8:**
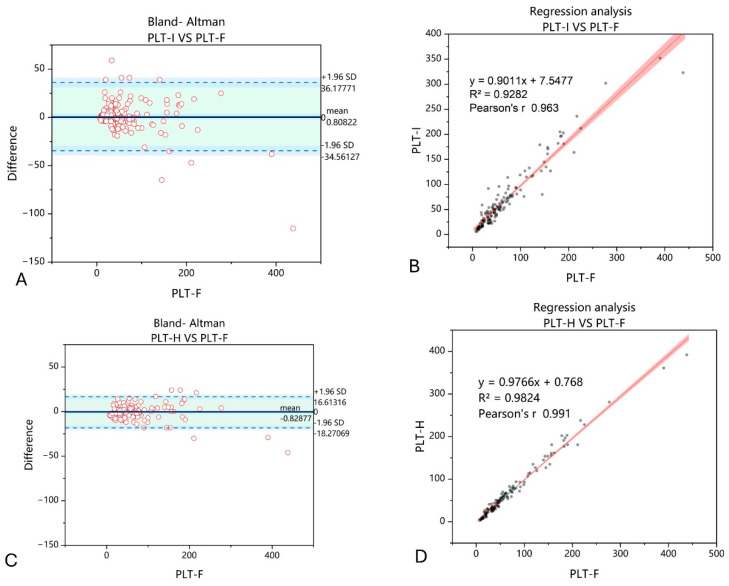
Comparison of PLT-I and PLT-H methods with regard to PLT-F in samples with MPV > 13 fL. Bland–Altman chart for PLT-I vs. PLT-F method (**A**) shows wider dispersion of results, with a mean difference of 0.81 compared to −0.83 obtained comparing PLT-H and PLT-F methods (**C**). The correlation of the obtained results increased when the PLT-H method on the Mindray BC6200 device was applied (**D**) compared to PLT-I (**B**). *n* = 146; PLT-I—platelet count measured with impedance method; PLT-H—platelet count measured with hybrid method; PLT-F—platelet count measured with fluorescence method.

Finally, we decided to find out whether PLT-H is also efficient in counting large platelets. We extracted samples from the study group (*n* = 146) with a mean platelet volume (MPV) of > 13 fL. In these samples, correlation between PLT-H and PLT-F was definitely higher than between PLT-I and PLT-F. It also influenced the dispersion of differences between PLT counts obtained with PLT-H, PLT-I, and PLT-F (Figure 8).

## 4. Discussion

We found that hybrid platelet measurement allows for obtaining more accurate platelet count results compared to the impedance method in samples with anemia.

Interferences in platelet counting are a widely known problem in hematology. Small red blood cells, RBC fragments, white blood cell fragments, or even lipid particles may affect PLT counting when blood samples are analyzed with the most basic and common impedance method. This generates laboratory errors, leading to inaccuracy and improper interpretation of test results [19,20].

The most common interference in PLT counting comes from microRBCs and schistocytes [14]. Deng et al. showed that a high probability of affecting the platelet count is found when the mean corpuscular volume (MCV) of RBCs is lower than 73.5 fL. In their study, the correlation between PLT-I and PLT-F in the presence of microcytosis was significantly worse than in samples with MCV > 73.5 fL (r = 0.877 vs. r = 0.999) [17]. Other studies also showed that the impedance method falsely elevates the PLT count [21,22]. To our surprise, we did not see such a difference between PLT-I and PLT-F that should be corrected by PLT-H. This could be explained by the small number of samples included in the analysis (*n* = 39) but also by the level of microcytosis that could differ in both studies. Interestingly, Chen et al. showed that it is not MCV that makes the PLT-I method unreliable but the absolute count of microRBCs, since the second parameter measured by PLT-I method on the Sysmex XN series analyzer enforced the performance of a reflex test with the PLT-F method (OR 0.94 for MCV vs. OR 2.51 for microRBC count) [22].

In our study, we observed an improvement in PLT count in samples with an increased amount of schistocytes (mainly due to mechanical shredding of RBCs in patients with artificial valves) when the PLT-H method was applied compared to the PLT-I method. The presence of fragmented RBCs was also previously recognized as a factor that falsely elevates the PLT count obtained by the impedance method of analysis [22,23]. Barros Pinto et al. reported an elevated platelet count in a patient on venoarterial extracorporeal membrane oxygenation (VA-ECMO) due to an elevated number of schistocytes [24]. Spurious thrombocytosis has also been reported in a patient with severe burns [25]. In case of the presence of schistocytes in a blood sample, either expressed as an increased automatically reported fragmented RBC count or their presence confirmed in a blood film, special attention should be paid to reliable platelet counting, since false PLT count elevation may lead to the omission of real thrombocytopenia (i.e., as a key feature of thrombotic microangiopathy) and misdiagnosis that will delay introduction of proper treatment [26].

Rare interference in PLT count may arise from WBC debris found in a blood sample from patients with leukemia [27,28]. We observed such a situation in one analyzed sample from the second study group (anemia due to hematological diseases) and found that, similar to the fluorescence method, the hybrid method allowed us to eliminate this error. PLT-I showed a falsely elevated PLT count compared to the PLT-F method (70 vs. 49 G/L), while the hybrid method showed the same PLT count as the PLT-F method (50 G/L). Even though a significant improvement in the reported PLT count was indicated by the PLT-H method, it was just one sample. Thus, further observations are needed to confirm that this method allows for the elimination of this sort of interference in all samples from patients with hematological neoplasms.

We showed that the PLT-H method is more accurate than the PLT-I method in anemic samples, which aligns with the observation made by Cai et al. They found that the hybrid method not only reduces the risk of obtaining false PLT count results due to the presence of interfering factors but also strongly correlates with the reference immunological method of PLT measurement [15]. Similar observation was made by Ye et al. [14]. In the present study, as a reference, we used the PLT-F method, which is the most accurate method available on automated hematology analyzers, and we concluded, similarly to the previously mentioned study, that both the PLT-H and PLT-F methods showed excellent agreement, with Pearson’s r ranging from 0.991 in anemic samples with large platelets to 0.999 in samples from patients with anemia caused by affected RBC production due to hematological neoplasms.

Large platelets in samples analyzed with the impedance method may not be incorporated in the final result, since only particles of less than 30 fL are counted with this channel [29]. When large platelets appear, they are flagged, and their presence is confirmed in a manual smear by routine. As PLT-H measures platelets larger than 10 fL in the differential channel, all—even giant platelets—are taken into account. Here, we show that Pearson’s r of PLT-H with PLT-F in samples with large platelets (>13 fL) is better than between PLT-I and PLT-F (R of 0.991 vs. 0.963). Surprisingly, Ye et al. obtained different results, since the linear correlation R^2^ between PLT-I and immunological PLT assessment was 0.97, and between PLT-H and the immunological method, it was 0.95 [14]. The difference may result from the number of included samples and maximal volume of PLTs; however; these were not defined.

Reliable platelet counting in patients with anemia might be challenging due to several factors associated with RBCs that may interfere with the most basic impedance method of platelet measurement. In this study, we included samples from patients with anemia of various causes, as different interferences in platelet (PLT) measurement may arise depending on the etiology of decreased hemoglobin concentration.

Group A consisted of patients with anemia resulting from non-hematological disorders—primarily liver failure, sepsis, and diabetes. The etiology of anemia in these cases is multifactorial, including malnutrition, splenomegaly due to portal hypertension, hemolysis, and coagulation insufficiency. Alcohol-related liver disease contributes to anemia through direct bone marrow toxicity and deficiencies in essential nutrients needed for red blood cell (RBC) production due to poor intake and absorption. Moreover, chronic inflammation can disrupt erythropoiesis, as proinflammatory cytokines reduce iron availability [30]. Since platelets are essential for maintaining hemostasis, and the factors affecting RBC production also influence PLT production, accurate PLT counting in such patients is crucial—especially considering that microcytes from iron deficiency and schistocytes caused by hemolysis may falsely elevate PLT counts. Furthermore, the PLT count may influence outcomes in liver transplantation; therefore, obtaining reliable PLT information allows for appropriate safety measures before surgery. Here, we show that PLT-H measures the PLT count more accurately than the impedance method, demonstrating the applicability of this approach.

Group B included patients with hematological malignancies or aplastic anemia. These individuals develop secondary hypoplastic anemia either as a result of the primary disease, its treatment, or due to chronic inflammatory conditions [31]. Such patients frequently experience severe thrombocytopenia and are at high risk of life-threatening bleeding. Reliable PLT counting is a matter of life or death, as delayed diagnosis increases the risk of intracerebral hemorrhage and premature death. From this perspective, not only the improved accuracy of PLT-H over PLT-I is important but also the precision in assessing the PLT count in thrombocytopenic samples, as demonstrated in our study.

A similar observation was made in Group C, which included patients with non-hematological malignancies. In these patients, anemia is mainly therapy- or chronic-inflammation-related [31]. Although they rarely develop life-threatening thrombocytopenia compared to patients with hematological malignancies, accurate PLT measurement allows for better monitoring of their general condition and more effective medical intervention when necessary.

As mentioned above, somewhat unexpectedly, we did not observe an improvement in PLT counting when using PLT-H instead of the PLT-I method in Group D—patients with microcytic anemia, primarily due to iron deficiency. Interference from microcytes in PLT-I measurement is one of the most commonly reported issues [9]. Although iron deficiency may falsely increase PLT counts measured by the impedance method, it is important to note that this condition also stimulates increased platelet production through expansion of megakaryocyte progenitors, accelerated megakaryocyte differentiation, or erythropoietin mimicking thrombopoietin activity [32]. Thus, accurately distinguishing between small red blood cells and platelets is essential to determine the true PLT count in patients with iron-deficiency microcytic anemia.

We also address the findings from Group E—patients with anemia due to cardiovascular conditions, including those with artificial heart valves. This group is unique in that there is both a high risk of intravascular hemolysis with schistocyte formation [33], which may interfere with PLT measurement, and frequent use of antiplatelet therapy aimed at reducing premature platelet activation [34]. Reliable PLT measurement—including large platelets—is essential for therapy adjustment and monitoring. Platelets play a central role in the pathophysiology of acute coronary syndromes, where their activation and aggregation contribute to thrombus formation following plaque rupture [35]. Moreover, patients with cardiovascular diseases such as myocardial infarction or heart failure have been shown to exhibit an increased proportion of large platelets, often consisting of immature platelet fractions that are more enzymatically and metabolically active than mature platelets [36]. Here, we demonstrate that PLT-H not only improves the accuracy of PLT counts in this patient group compared to the impedance method but also significantly enhances reliability when large platelets are present. Therefore, applying this methodology is beneficial for proper evaluation of hematological parameters in cardiological patients.

## 5. Conclusions

Platelet counting using the impedance method is susceptible to interference and should be interpreted with caution. Thus, additional methods for PLT counting should be applied, like optical or fluorescence methods, to provide as much accurate PLT count information as possible. However, the application of these methods carries additional costs that arise from the necessity of using expensive fluorescent reagents. Here, we showed that a novel hybrid method of platelet measurement that uses two basic channels commonly used for complete blood count assessment is worth considering as a cost-effective solution that allows for obtaining reliable PLT counts in anemic samples of different etiologies, which does not deviate from fluorescence measurement.

## Figures and Tables

**Table 1:** Number and primary classification of samples included in the study.

Group	Cause of Anemia	Number of Samples
A (non-hematological metabolic disorders)	Alcoholic liver disease/ HCV infection/Cirrhosis/Sepsis/Qualification for liver transplantation/Primary biliary cholangitis/Diabetes	197
B (hematological cancers/disorders affecting WBC, RBC, and PLT production)	Aplastic anemia/Leukemia/Lymphoma/Multiple myeloma/Myeloproliferative neoplasm	193
C (non-hematopoietic-related solid tumors)	Hepatocellular carcinoma/Colon cancer/Gastric cancer/Lung adenocarcinoma	90
D (microcytic anemia)	Iron deficiency anemia/Thalassemia/Chronic blood loss	39
E (non-inherited hemolytic anemia)	Anemia associated with cardiovascular events	29
F (acute blood loss anemia)	Surgery/Acute gastrointestinal bleeding	19
G (ITP)	Idiopathic thrombocytopenia	8
H (TTP)	Thrombotic thrombocytopenic purpura	2

Among all samples, 413 samples were thrombocytopenic with PLT count of less than 100 G/L, including 305 samples with PLT < 50 G/L.

**Table 2:** Proposed acceptable criteria for deviation from PLT-F count in samples measured with PLT-I and PLT-H methods.

Obtained Value	Absolute Deviation	Percentage of Maximal Target Value
0–10 × 10^9^/L	±5 × 10^9^/L	50%
10–20 × 10^9^/L	±8 × 10^9^/L	40%
20–50 × 10^9^/L	±10 × 10^9^/L	20%
50–100 × 10^9^/L	±20 × 10^9^/L	20%

## Data Availability

The data presented in this study are available on request from the corresponding author. The data are not publicly available due to ethical reasons.
